# Digital-Based Healthy Bra Top Design That Promotes the Physical Activity of New Senior Women by Applying an Optimal Pressure

**DOI:** 10.3390/ijerph18094651

**Published:** 2021-04-27

**Authors:** Kuengmi Choi, Jungil Jun, Youngshil Ryoo, Sunmi Park

**Affiliations:** 1 Department of Fashion Design, Dong-Seoul University, 76, Bokjeong-ro, Seongnam-si 13117, Gyeonggi-do, Korea; orogi1961@hanmail.net (K.C.); junjungill@hanmail.net (J.J.); ysryoo51@hanmail.net (Y.R.); 2 Department of Fashion Design, Konkuk University, 268 Chungwon-daero, Chungju-si 27478, Chungcheongbuk-do, Korea

**Keywords:** healthy ageing, human body modeling, clothing pressure, virtual fitting, 3D scan data, flattening 2D pattern, clothing fit

## Abstract

A bra use can reduce physiological and physical functions because of clothing pressure, which can be a problem for new senior women starting to lose physical function. The present study presents a bra top design development method for promoting new senior women’s physical activity by identifying problems related to bras’ effects on women’s health and minimizing clothing pressure. The analysis utilized the 3D scan data of 42 adult women (age range: 50s) from the 5th Size Korea Project. Bra top design elements were extracted based on new senior consumers’ needs. We developed an average wireframe reflecting the new senior’s physical characteristics, and a standard body form was developed through surface modeling. To produce a consumer-oriented bra with a body shaping effect and reduced clothing pressure that would not affect physical activities, a three-dimensional pattern was developed applying an optimal reduction rate of 80%. To verify the bra’s adequacy for the body form of new senior women, two market-available bras were selected and fit-compared to the developed product. The developed bra received higher expert appearance evaluation and 3D virtual clothing evaluation scores. This study is significant because by using virtual fitting technology, it provides foundational data to quantify the quality of fashion products.

## 1. Introduction

The world’s life expectancy is increasing due to economic, social, and medical advancements; as living standards have increased, the population of the aged 65 and over (more than 7% of the total population) has increased. According to a report by the World Health Organization (WHO), average human life expectancy could increase from 66 in 1997 to 73 in 2025, and the proportion of elderly people (aged 65 and older) may increase from 7% (1997) to 10% (2025). Moreover, the WHO predicts that by 2025 the number of countries with an average life expectancy of over 80 years will reach 26 and that no country will retain an average life expectancy of 50 years or less [1]. The demographic structure of age distribution is changing and the consumption market is expanding with a focus on the elderly and women; consequently, a “silver” age group is emerging as a new consumer segment [2]. The definition and age criteria for the elderly vary by law and by the researcher [3]. When considering the elderly as consumers and analyzing their purchasing activities or consumption tendency, they are sometimes distinguished from the existing seniors by calling them in their 50s to 60s by using the term “new senior” [4,5]. Unlike past elderly age groups, the new senior age group, led by the baby boomer generation, is socially active and is not hesitant to spend considerable time and money on aspects of themselves including appearance and health [6,7]. Sherman et al. [8] predicted that the market for the elderly would grow into a leading consumer market in the 21st century and defined the elderly as “young-again-rich.” As such, the new senior age group, which pursues a lifestyle different from that of the existing elderly group, has significantly influenced the apparel and fashion market. Further, a need to form a market for new senior women is also emerging in the underwear market [2].

Humans, as organisms, are born, grow, and develop, and they change as they age with the progress of the biological aging process. Aging can be defined as a gradual change that occurs in cells, tissues, organs, or throughout all parts of an organism over time [9]. With the aging process, women experience height decreases, and their backs become bent owing to bone and muscle atrophy; furthermore, the subcutaneous fat decreases, resulting in a thinner body and sagging breasts [10]. As aging progresses, the breast changes the most in terms of form. Thus, among elderly women, the demand for a bra that can shape the body form is increasing. A bra supports the breast and gathers it inside the cup to help maintain an ideal shape. Concerning body shaping, wearing underwear that puts too much pressure on the body can cause changes in the body shape and also trigger stress or health disorders [11,12]. Thus, wearing underwear that fits one’s body type and size is very important [13,14]. Whereas new senior women are active and interested in maintaining their appearance, especially with regard to underwear, few products, including most market-available bra top products targeting new senior women in their 50s or older, have reflected new senior’s body shape and needs; accordingly, complaints from new senior consumers about underwear are on the rise [15,16].

The clothing pressure occurs when clothing and the human body come into contact. It refers to the pressure that a body wearing clothing receives from the clothing weight and tightening or pulling resulting from clothing fit [11,12]. When wearing clothing that strongly compresses the human body, such as a bra, corset, or girdle, the human body receives high pressure on the body, and the clothing pressure varies depending on the posture, movement, and characteristics of the material [17]. Therefore, clothing pressure is a major design factor in designing close-to-body clothing, such as underwear. An appropriate range of clothing pressure can improve clothing fit and, thus, facilitate physical activity. It does not only improve work efficiency but also helps to maintain and promote health [18,19,20]. On the other hand, excessively high clothing pressure may cause indigestion or duodenitis by altering the location and form of the body’s organs. It may lead to diseases, such as backache, edema, and growth disorders. Wearing high-pressure clothing for long periods decreases blood flow and may lead to muscle fatigue and impaired blood flow [11,17,21,22,23]. According to previous studies [12,24,25,26,27] that examined the effects of bra use-related clothing pressure on the physiological response of the human body, bra straps and wires were the biggest contributors to clothing pressure; furthermore, wearing a bra that compresses the breast area for a long period of time puts placed pressure on the heart and affected blood circulation and lymph flow, as the bra surrounds the breast horizontally, it interferes with breathing and the circulation of lymph and blood flow. Furthermore, studies [28,29,30] have found that it stiffens surrounding muscles and causes neck and back pain. Previous studies have suggested that the clothing pressure was relatively high in compression-style sports bra tops with reinforced motion function, thus involving a high risk of causing physiological disorders when worn for long periods. In addition, bra tops with hypoallergenic function had low clothing pressure and were found to be comfortable; however, they had a poor body shaping function. Therefore, a comfortable bra top that affects shaping a breast form that has sagged due to aging and the appropriate clothing pressure level for new senior women should be developed.

Clothing fit measurements include qualitative and quantitative methods. Although a quantitative evaluation method based on objective data is desirable, obtaining such data is not easy [31,32]. Therefore, in the apparel field, clothing fit is often measured by relying on visual and qualitative evaluation. The 3D body scanning technology offers some technical advantages: a human body form can be realized as 3D surface data, and a non-contact precision dimension measurement becomes possible. Thus, this technology has been used to design products in various industries, including the clothing industry [32,33,34,35,36]. In particular, 3D virtual fitting technology makes it possible to measure clothing fit more objectively and visibly by providing data such as the degree to which the clothing can be deformed by an external force or the degree of tightness of the clothing [37,38,39,40]. Studies aiming to use virtual fitting technology to measure clothing fit have been actively conducted in the apparel field [34,39,41,42,43,44]. It is necessary to verify whether this technology can suitably replace the existing fitting tests, which use actual human bodies, and whether this can be achieved by targeting various age groups and various clothing types so that the virtual fitting technology can be recognized as a measurement tool to evaluate the quality of the clothing.

Lim and Cho [45] stated that female consumers in their 50s have different characteristics from female consumers in their 60s or older in terms of areas where they feel uncomfortable when wearing bras and the type of bra they prefer. Choi, Ryu, Kim, and Jun [46] argued that because women in their 50s have different body shape characteristics than women in their 60s or older, bras that reflect the body shape characteristics of women in their 50s should be developed separately. In this study, women in their 50s were selected as subjects. Wearing a high-pressure bra for long periods can cause discomfort and physiological disorders. Nevertheless, new senior women still want to look attractive and wear underwear that can shape their body form. This study aimed to develop a bra top that can promote healthy physical activity among new senior women by minimizing the clothing pressure and also provide an aesthetically pleasing body shaping effect.

The objectives of this study are as follows:

1. To extract bra top design elements that reflect the needs of new senior consumers.
2. To use the 3D modeling technology to develop a virtual human body model that reflects the characteristics of new senior women’s body form.
3. To develop a three-dimensional pattern while considering an optimal clothing pressure that will not severely affect new senior women’s physiological function and to simultaneously provide a consumer-desirable body shaping effect.
4. To verify whether the use of 3D virtual fitting technology is suitable as a tool for measuring the fit of the bra top of a new senior woman.

This study’s development of a bra top that simultaneously provides comfort and a body shaping effect is expected to encourage new senior consumers’ external and psychological confidence, thereby helping them live healthy and active lives during retirement. Moreover, this study aims to develop a virtual model through the multi-faceted body form analysis using 3D scan data; this is expected to help vitalize the customized underwear market for new senior women, which, in turn, could help prepare for the emergence of a personalized market through the online market in the future. Regarding the measurement of clothing pressure, which is the most important factor in new senior women’s clothing life, this study verified whether the virtual fitting technology could be used as an objective tool for measuring the clothing fit in the future. This technology measures the fit of fashion products that are difficult to evaluate quantitatively. The introduction of a virtual fitting evaluation technology that is based on these objective data could contribute to improving the quality of clothing products.

## 2. Materials and Methods

### 2.1. Extracting Design Elements That Reflect Consumer Needs

The term “bra top” is an abbreviation of “brassiere top,” and is an item of women’s clothing that looks like a *bra.* It is functional clothing that enhances the aesthetics of the breast by shaping its form and minimizing breast movement by containing the breast with an appropriate level of pressure [16,47,48]. The three elements that form a bra are cup, strap, and wing. In addition, there are front panels, breast lines, wires, hooks, eyes, and adjusters. The cup provides overall protection and shaping for the breast; the strap assists in stabilizing the breast; the wing provides pressure for the muscles in the armpit area [16]. Depending on the cup shape, bras are classified into full cups, which cover the entire breast, 3/4 cups, which cover 80% of the breast, 1/2 cups, which cover only half of the breast, and triangle cups, which wrap the breast in a triangular shape. Furthermore, depending on the material that forms the cup, bras can be classified into mold type, non-woven fabric type, and single-layer type. Based on wing form, bras are classified into straight type, U shape type, vest type, and running type [13,49,50].

Some previous studies on wearing characteristics and requirements of a bra surveyed female consumers in their 50s or older [45,47,51,52,53,54]. These studies showed that women in their 50s wore bras to prevent exposure and breast movement; they wanted a functional bra that provided comfort and also a breast gathering effect to some extent. In terms of specific design elements, they preferred designs that were easy to put on and take off, fixed straps, and shapes that covered the entire breast. They felt uncomfortable wearing any purchased bra that did not match their breast shape; in particular, most of their discomfort depended on the bra cup fit. It was found that women in their 50s prefer bra cups that help them aesthetically correct the shape of their saggy breasts and balance their bodies. They also showed that they prefer a design that can cover the flab on the sides and back caused by aging.

As for the effect of bras on new senior women’s body form, new senior women tended to wear bras selectively—for example, only briefly when going out—because the bras were stuffy and uncomfortable in motion if worn continuously [55,56]. In particular, there were several complaints regarding feelings of pressure caused by the tightening of the strap by the wire, which was inserted into the bra, or the load on the breast, and there were also many complaints regarding stiff shoulders and breathing discomfort [55,57,58].

Based on this previous research, this study aimed to design a bra top with the following three design development objectives. First, the designed bra should completely adhere to the human body and not interfere with its movement. Second, it should shape the body form, improve aesthetics, and enhance the body’s balance. Third, there should be no excessive restraints caused by the clothing pressure.

### 2.2. Development of a Virtual Human Body Model Reflecting the Morphological Characteristics of the Body

To increase the satisfaction of a given consumer group that is being targeted by a clothing manufacturer for clothing fit, the manufacturer uses a body form or fitting model that expresses the body shape of the target group accurately [34]. The body form is the standard for clothing manufacturers to design clothing. It is used as an important production factor in the apparel industry and in processes including the design development, pattern, sample production, and the inspection stage. Like the body form or fitting model used by clothing companies, this study’s developed virtual human body model is used to design clothing in a virtual space or based on a certain fit measurement.

#### 2.2.1. Developing a Standard Body Form for New Senior Women

**Figure 1:**
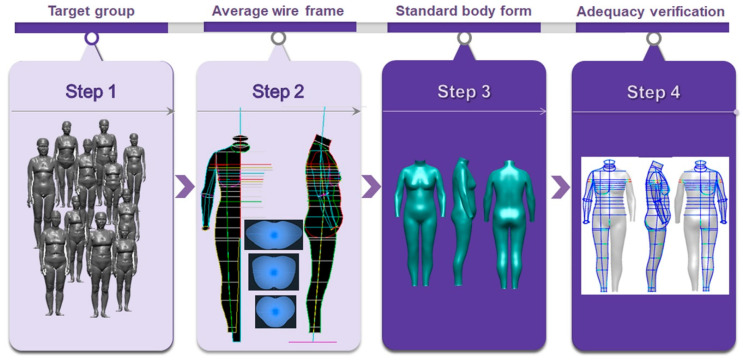
The development process for standard body form of new senior women.

This study developed a virtual human body model to reflect the body form characteristics of new senior women; this tool was developed to create a digital-based healthy bra top design for new senior women. The virtual human body model was developed through the following process: the target group was selected, and an average cross-section was created using the 3D body form scan data belonging to the group. Next, an average wireframe was developed by combining the average cross-section. By performing surface modeling, a standard body shape, which is highly consistent with the developed average wireframe, was developed. Finally, the adequacy of the developed standard body form was verified (Figure 1).

##### The Target Group

Among the size specifications for new senior women’s bra tops, those who used the medium size were selected as the target group. Choi, Ryu, Kim, and Jun [46] developed a size specification for bra tops for 350 adult women aged 50–59 years. They developed a size specification based on the characteristics of body form and lifestyle for new senior women and, as a result, presented size 80A as the medium size. The medium size corresponds to the high-frequency section as a result of cross-analysis of the size of the bra cup at 2.5 cm intervals and the size of the underbust circumference at 5 cm intervals. Thus, the present study selected size 80A for the target group. The 80A size means that the underbust circumference is 80 cm, and the cup size is A cup, that is, the difference between the bust circumference and the underbust circumference is within 10 cm. This study used the scan data of 42 adult women in their 50s that used the 80A size from among the data of the 5th Size Korea Project [59]. In this study, when selecting the target group, the proportion of the body, the balance of the torso, and the lateral posture were considered. In other words, subjects with similar human dimensions, body shape, and lateral posture were selected for the study.

##### Average Wire-Frame Generation

To create an average wireframe, a human form curve was made based on the 3D scan data of the target group. The Rapidform XOR3 software (Inus Technology Inc., Seoul, Korea) was used to create the curve collection. To create the average cross-section, the right cross-section of the scan data of the target group was taken. Regarding the collected section, an average section was created using the average of the length values by measuring the length of the curve from the center to the section at 1-degree intervals. After that, the cross-section was symmetrically aligned. A total of 37 sections, including 28 cross-sections, 4 longitudinal sections, 3 body axes, and 2 breast form sections, were created. Then, they were assembled to complete the average wireframe. By using body axis data that indicated the posture of the human body (i.e., the neck angle, upper body axis angle, and lower body axis angle) when constructing the average wireframe, this study aimed to reflect not only the body form characteristics of new senior women but also the characteristics of the posture. The AIP 2008 (AutoCAD 2008) software (AutoDesk Inc., San Francisco, CA, USA) was used to generate the average wireframe.

##### Standard Human Modeling

The standard body form for new senior women was created by modeling a virtual human body, which most closely matched the average wireframe and had a natural surface; this step was carried out while referencing the average wireframe developed in the previous step. The surface modeling using the free form deformation (FFD) method was conducted using 3D-Max software.

##### Form Conformity Verification

The conformity of this study’s developed virtual model to the average wireframe was verified. The rapid form XOR3 software was used for conformity verification. The morphological adequacy was analyzed by calculating the distance deviation between the average wireframe and the virtual modeling form. In this study, the tolerance criteria proposed in ISO 20685–1 were used to verify form conformity. ISO 20685–1 [60] provides a tolerance range of ±9 mm for large circumferences and ±5 mm for segment lengths.

#### 2.2.2. 3D Molded Bra Cup Development

**Figure 2:**
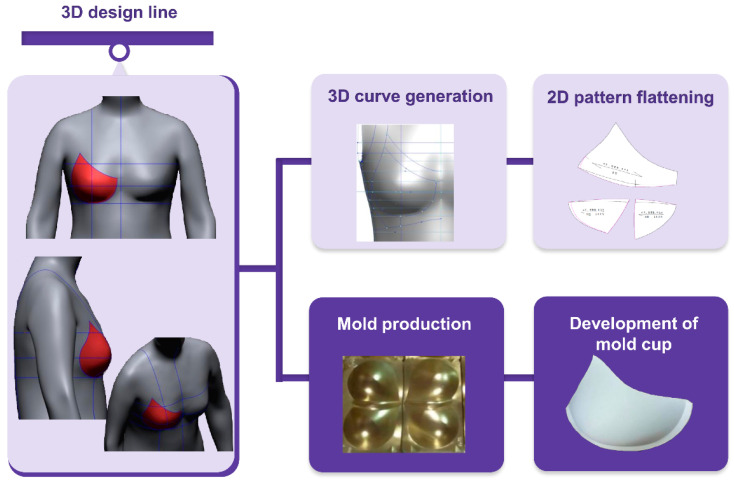
3D molded bra cup development process.

This study developed a molded bra cup to develop a bra top, which did not only reflect the characteristics of new senior women’s body form but also offered a body shaping effect and sufficient cover for the breast. The molded bra cup is designed in a shape that can correct the shape of the breast sagging due to aging and gathers both breasts in the middle. After drawing a design line for molded cup development on the 3D standard form, the 3D form was developed using a triangular mesh and then developed again using a 2D flat pattern. Further, a 3D molded bra cup was finally developed by transforming the 3D molded cup form into a mold. Figure 2 shows the development process for the molded bra cup.

### 2.3. Developing Three-Dimensional Patterns That Consider Optimal Clothing Pressure

After creating a standard virtual model that represented the body form of new senior women, this study developed a bra top pattern by developing the surface of the virtual model in a two-dimensional pattern. A bra top produced in such a way is a 3D surface of the virtual model that is unfolded in 2D; therefore, it does not have a body shaping effect. To produce a shaping effect that supports and gathers the breast, the 2D pattern must be reduced. The pressure on the breast that the bra top supports varies depending on the reduction rate. The question of what reduction rate should be applied to the 2D pattern is very important as it is linked to the comfort of the clothing. This is because a high clothing pressure improves the function of supporting and gathering the breast; however, it constrains a wearer’s physical activity and impairs physiological function. On the other hand, if the clothing pressure is low, breathing is comfortable, and the fit is good, but the function of shaping the breast is limited.

This study defined the optimal clothing pressure as the clothing pressure that helps to shape a consumer-desirable body form and does not pose any problems for the physical activity or physiological function of new senior women. Cho [61] aimed to find the optimal pressurization rate for the bra top; to this end, a human experiment was conducted. After making lab coats of molded and nonwoven bra tops, a fitting test was conducted for women in their 50s by applying reduction rates of 90%, 80%, and 70%, respectively. Then, clothing pressure measurement, evaluation of subject’s wear sensitivity, and appearance evaluation by experts were conducted to analyze the objective data on clothing pressurization. The clothing pressure was measured at 8 locations on the bra top, and clothing pressure was measured and analyzed for each posture. As a result, the optimal clothing pressure was observed when the pattern reduction rate of the molded bra top was 80%.

Based on the results of previous research [61], this study intends to develop a three-dimensional pattern that considers the optimal clothing pressure by applying a pattern reduction rate of 80%. In addition, it aims to verify the possibility of product production through real-world production of the bra top.

### 2.4. Evaluating Clothing Fit

To verify the level of adequacy of this study’s developed bra top for the body form of new senior women, two market-available bra tops were selected and compared with the product developed in this study. Commercially available products that had a design similar to the bra top developed in this study were selected among those that had the same size specification and a similar fiber mixture rate. The design line and clothing construction, seam, and dart of the 2 comparison-target bra top products were analyzed. The fitting test was conducted in two ways: an expert’s appearance evaluation and a virtual fitting evaluation.

#### 2.4.1. Experts’ Appearance Evaluation

The selection criteria for the experts were as follows: those who had obtained at least a master’s degree in apparel studies and had more than 10 years of work experience in the apparel field. For the expert appearance evaluation, data were collected by requesting the participants to respond to a total of 8 questions on a 5-point Likert scale. For data analysis, the reliability of the questionnaire answers was analyzed using the statistical package SPSS 25.0 for Windows (IBM, Armonk, NY, USA).

#### 2.4.2. Objective Evaluation Based on the Virtual Fitting Technology

A virtual body and digitized clothing data are necessary for performing a 3D virtual clothing evaluation. This study’s developed bra top did not require digitization because the digitized clothing data were already available; however, the data of two market-available bra top products required digitalization. Digital clothing data were secured by disassembling the 2 market-available bra top products and digitizing each pattern piece. To secure digital clothing data, YUKA CAD, which is one of the Apparel CAD Systems, was used. The secured digital clothing pattern was applied to the standard virtual body form of a new senior, and virtual fitting was performed. The software used for virtual fitting was CLO 3D. As a 3D fashion design software program, the CLO 3D program simulates the actual clothing in 3D by digitally realizing the material and physical properties of the fabric. This program provides data that can objectively determine the clothing fit by judging the range of pressure distribution between the avatar and the clothing and the distribution of contact points.

In this study, strain map and fit map were analyzed to evaluate the 3D garment’s fit. A strain map is an index that indicates the degree to which clothing is deformed by an external force. The strain rate is expressed as a percentage. It is represented by eight color distributions. A case in which there is significant strain is expressed in red, and a case in which there is no strain is expressed in blue. When there is a moderate strain between the two cases, it is expressed in the gradation of the two colors. A fit map indicates the tightness of clothing. If clothing is too tight to wear, it is marked in red, if it is very tight, it is indicated in orange, and, if it is slightly tight, it is indicated in yellow [62,63].

## 3. Results

### 3.1. A Bra Top Design That Reflects Consumer Needs

This study extracted the design elements of the bra top based on previous studies and literature. The design elements of the bra top were extracted into four factors based on the preferences and requirements of new senior consumers and the effect of clothing pressure on the human body. These factors are aesthetics, wearing habits, physiological characteristics, and physical characteristics.

The bra top was designed in the form of a molded bra cup to reflect aesthetics and to beautifully shape breasts that may sag because of aging; a full cup covered the entire breast. Considering the wearing habits, it was designed to facilitate easy donning and removal, and it can be worn in everyday life and during light exercise. To minimize the pressure felt on the shoulder, the contact position of the shoulder and strap was adjusted, and the contact surface was widened by increasing the size of the strap, thereby reflecting the physiological characteristics of new senior women. In addition, the wire was removed to ensure that it would not interfere with lymph and blood flow. Lastly, the bra top was designed to reflect the shape of the new senior women’s body and breasts, thereby reflecting the physical characteristics. Using the cutting line considering the flow of muscles, exercise functionality was improved. In addition, the wing of the bra cup was designed to be wide such that the fat from aging can be covered.

**Figure 3:**
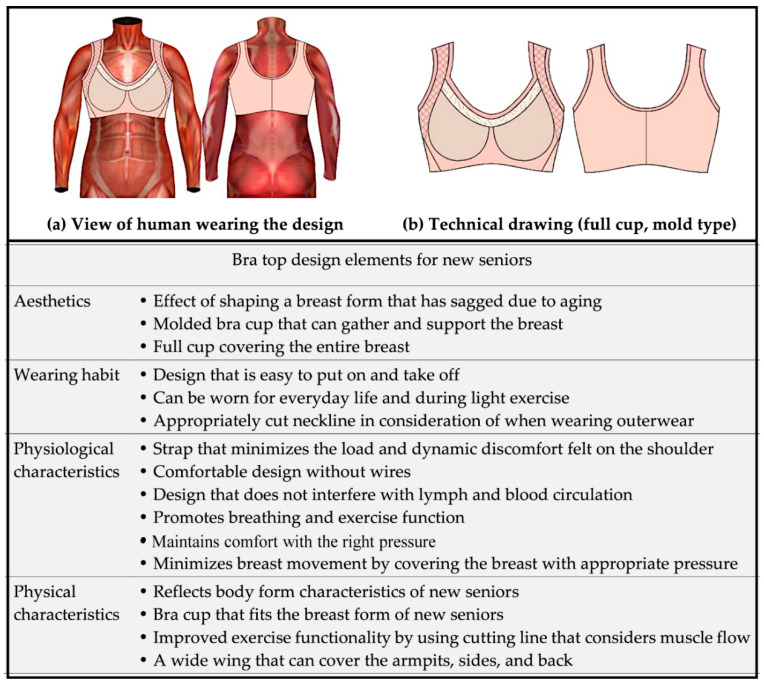
Results for a bra top design that reflects the needs of consumers.

Figure 3 shows the result of designing a new senior-targeted bra top that reflects design elements such as aesthetics, wearing habits, physiological characteristics, and physical characteristics.

### 3.2. A Virtual Human Model That Reflects the Morphological Characteristics of the Body

#### 3.2.1. Developing a Standard Body Form for New Senior Women

The average human dimensions of the target group were as follows: height, 1563 mm; weight, 56 kg; bust circumference, 898 mm; and under bust circumference, 798 mm (Table 1).

**Figure 4:**
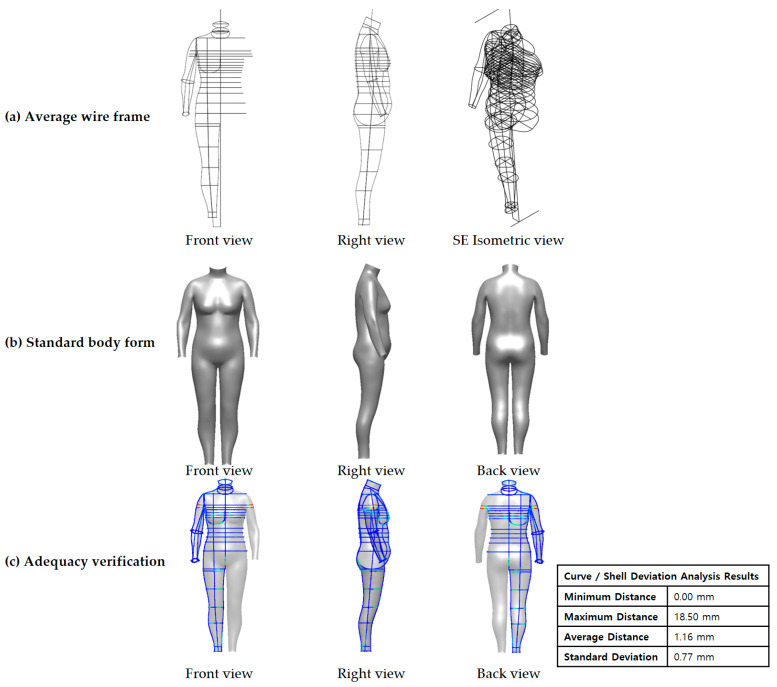
Results of development of standard body form for new senior women.

Figure 4a presents the results of creating an average wireframe using the 3D scan data of 42 adult women in their 50s. An average wireframe was completed by combining 37 average sections. The average wireframe reflected the average human dimensions of the target group and its physical characteristics. Moreover, since the average wireframe reflected the average values that indicated the posture of the side (for example, the neck angle, the upper body axis angle, and the lower body axis angle), it was found to reflect the posture characteristics of the target group. Figure 4b illustrates the results of modeling the standard human body form for new senior women based on the average wireframe. After verifying the form’s adequacy to confirm the level of similarity between this study’s developed virtual model and the average wireframe, the average wireframe and the virtual modeling form were found to have an average distance deviation of 1.16 mm (Figure 4c). These deviations fall within the tolerances provided by ISO 20685–1. When the distance between the curve and the 3D is close, it is expressed in blue. When the distance is further, it is expressed in red. The medium distance is represented by a gradient color. Thus, this study’s virtual human body sufficiently reflected the target group’s average human dimensions, morphological characteristics, and even lateral postures.

#### 3.2.2. 3D Molded Bra Cup Development

Table 2 presents the results of developing the 3D molded bra cup using the 3D standard form for new senior women. To verify any errors in the molded bra cup, the difference in length from the 2D flat pattern was analyzed. When the upper and lower sides and the width of the cup were compared, the upper side of the cup was found to have the largest error. The relative error was 7.57% and it was larger for the molded bra cup. This error occurs when manufacturing the molded cup from the mold.

### 3.3. Developing a Three-Dimensional Pattern That Considers Optimum Clothing Pressure

**Figure 5:**
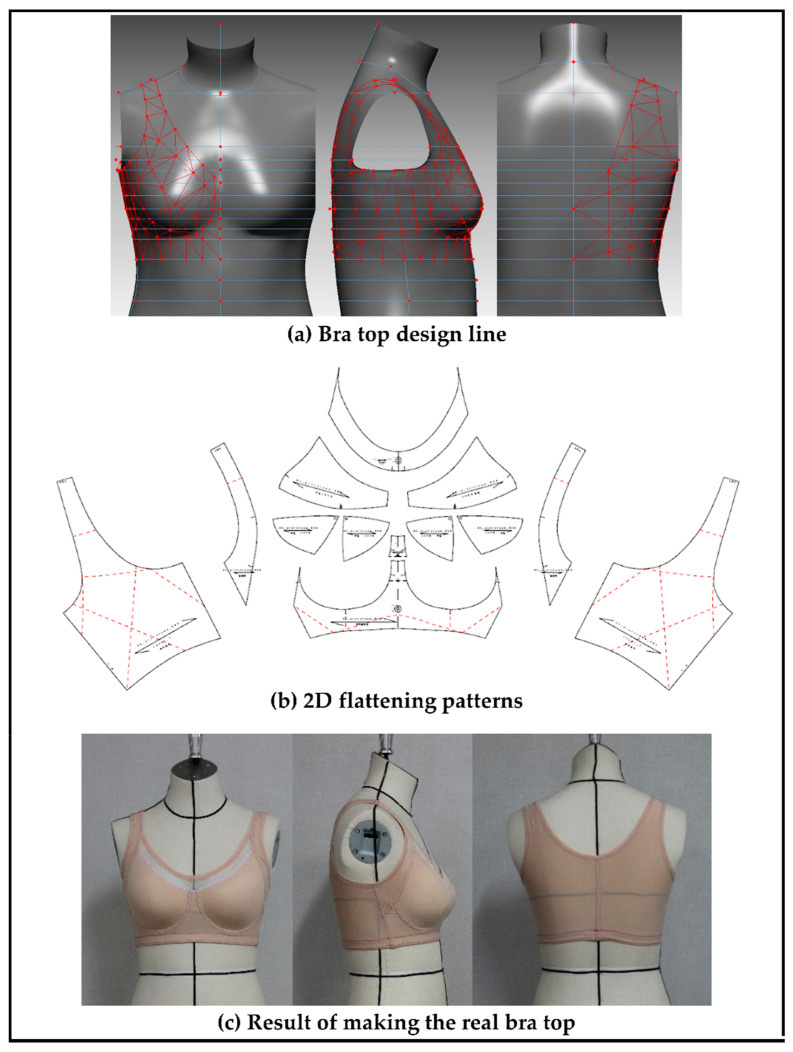
Development of a three-dimensional pattern considering optimal clothing pressure.

To develop a bra top with a body shaping effect and a clothing pressure that does not interfere with new senior women’s physical activity and physiological function, the present study set a reduction rate of 80% as the optimal clothing pressure. After drawing the design line of the bra top on the surface of the virtual human body model representing the new senior women, a triangular mesh curve was created. The 3D mesh curve was developed in a 2D flat pattern; after this, a final three-dimensional pattern was developed by reflecting the reduction ratio of 80%. Figure 5a,b show the results of creating the design line of the bra top and 2D flattening patterns by using the 3D virtual model. In addition, a real bra top was created using the pattern produced. This process confirmed the commercialization potential of the bra top design for the new senior women (Figure 5c).

### 3.4. Evaluating the Clothing Fit

To measure the fit of the bra top developed in this study, two market-available bra tops—that is, brands A and B—were selected for comparison. The technical drawing in Table 3 presents the results of analyzing the two bra top products, which were the comparison targets. Brand A was a sports bra top and had a molded bra cup; it was structured to be worn using a hook and eye in the form of a back closing. Brand B was also a sports bra top, had a molded bra cup, and a wide band at the bottom; it was structured to be worn by using snap buttons in the form of a front closing.

#### 3.4.1. Expert Appearance Evaluation

Expert appearance evaluations were conducted on two bra tops, which were the comparison targets, and this study’s developed bra top. After the reliability verification for expert appearance evaluation response, Cronbach’s alpha value was 0.749; the showed high reliability. Table 4 presents the results of the expert appearance evaluation. The evaluation results showed that this study’s developed product had an average point of 4.39, which is higher than brand A’s average point of 2.30 and brand B’s average point of 3.79. Particularly, the study-developed product received a markedly higher score than the other two brands in questions 1, 2, and 3, which sought information about the fit of the chest area. This finding can be attributed to the fact that the bra cup reflects the new senior women’s body form well and, thus, sufficiently covers the breast; it also fits the body well without lifting any parts.

In the fit evaluations for the shoulder and back, brand B and the study-developed product received almost similar high scores. This is consistent with the results of previous studies [47,64] showing that straight rather than X-shaped straps distribute the clothing pressure and do not impede physical activity. Further, wider wings on the bra were found to be associated with greater coverage for the fat on the back and a comfortable fit.

#### 3.4.2. Objective Evaluation Based on Virtual Fitting Technology

**Figure 6:**
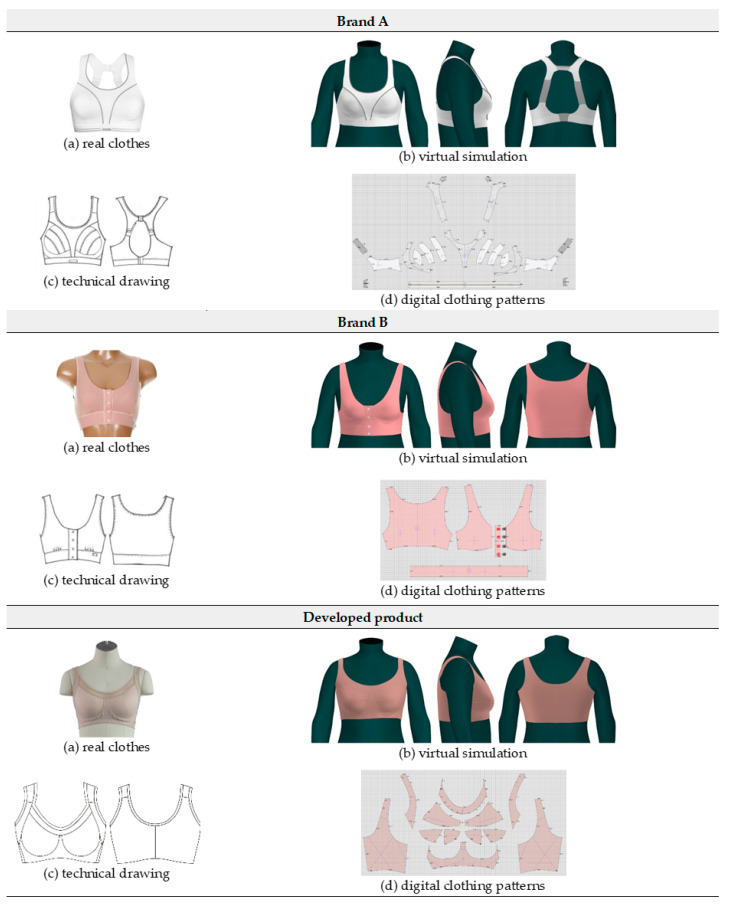
Results of virtual clothing simulation for virtual fitting.

Figure 6 shows the results of the simulation by placing the digitized clothes of three bra tops on the new senior virtual model. For objective evaluation of garment fit, strain map and fit map were analyzed.

Table 5 shows the virtual fitting result obtained using the Strain Map. A greater transformation effect of external forces on digitized clothes was expressed in red. In the case of the bra, the biggest issue was how to distribute the clothing pressure from the shoulder. This study’s developed bra top was found to have the least strain in the shoulder area compared to the other two brands. This finding could be attributed to the fact that structural design aspects, such as the position of the strap, the width of the strap, and the design line, were supported to distribute the load felt from the shoulder when developing this product.

Table 6 illustrates the virtual fitting result using the Fit Map. Fit Map provides more objective numerical data than the Strain Map. Concerning the frequency of cases where the bra was too tight to wear, Brand A had 17.0%, brand B had 39.7%, and the study-developed product had 2.9%. After comparing the tightness of the clothes, the developed product was confirmed to be excellent in this regard.

## 4. Discussion

High clothing pressure, which restrains the body, is aesthetically pleasing but it increases fatigue. Conversely, if the clothing pressure is low, the physical fatigue level decreases, but the appearance is not as beautiful [64,65]. This study involved the designing of a bra top to promote new senior women’s healthy retirement activities by minimizing body pressure while also offering the effect of shaping beautiful aesthetics. The study results were as follows.

First, the bra top design elements were extracted into four categories: aesthetics, wearing habit, physiological characteristics, and physical characteristics. This was achieved by comprehensively considering the preference and requirements of new senior consumers and the effects of clothing pressure on the human body. Park and Jang [64] proposed a bra product design for new senior women based on the results of a survey on bra-wearing conditions and design preferences. Heo [16] proposed the design of a bra for new senior women, focusing on aspects of functionality, such as pressure minimization, physical protection, and body type correction. The current study is different from other studies as a bra was designed to reflect the needs of new senior consumers and considering the aesthetic, physical, and physiological characteristics of new senior women.

Second, to develop a virtual human body model that reflected the characteristics of new senior women’s body form, size 80A was selected as the target group, and an average wireframe was developed by analyzing the scan data of 42 adult women in their 50s belonging to this group. By modeling a virtual human body, which had a natural surface and aligned with the average wireframe, a standard human body for representing new senior women’s body form was completed. Calculating the distance deviation between the average wireframe and virtual modeling form to verify the adequacy of the modeling results, the average distance was found to be 1.16 mm. Accordingly, this study’s developed virtual model was found to reflect the average dimensions and form of the target group as well as the characteristics of the side posture. Kim and Choi [10] used 3D scan data to categorize the body shape of new senior women; they argued that the development of clothing products for elderly women should reflect the morphological characteristics of their human bodies because of the increased age. Cha [66] reported that elderly women change their postures a great deal as they get older, so when developing a representative body shape for elderly women, it is necessary to consider the axis angle of the human body. Jeon, Park, You and Kim [65] developed a representative body shape of an elderly woman to design stereoscopic patterns that adhere to the body using 3D scan data. They aimed to design a product for preventing hip fracture, so they developed a representative body type based on hip protector size specifications. In the current study, a representative body type was developed considering not only the morphological characteristics of new senior women’s human body but also the body axis indicating the lateral posture. In addition, since the purpose of the current study was to design a bra top product, the body shape was developed based on bra top size specifications. The current study is different from other studies as the development of a molded bra cup with a body shape correction effect can provide data for the development of bras that increase the satisfaction of new senior female consumers. Zhang et al. [14] found out that the bra fitting of elderly women is deeply related to bra design features, and proposed an optimal design to solve the bra fitting problem of elderly women; wide straps at the top of the bra, wide straps at the front of the bra, adequate vertical cup length, increased gore height, fabric-applied strap back and rigid under band are recommended in bra designs for older women. The bra design they proposed is similar to the bra top design developed in this study.

Third, to produce clothing products through processes such as the shaping of underwear using 3D modeling technology, the process of reducing the pattern must be undertaken after developing the 3D surface in 2D. In this study, a three-dimensional pattern with the body form shaping effect desired by the consumers, and a clothing pressure that did not negatively affect new senior women’s physiological function were developed by selecting an optimal pattern reduction rate of 80%. Lee, Hong, and Lee [67] pointed out that designing patterns for the production of clothing products using 3D scan data and 3D technology can produce clothing that has a better fit and is more comfortable to wear than conventional pattern designs. They also argued that the clothing pressure varies according to the reduction rate of the 3D pattern and that the optimum clothing pressure has the effect of improving the wearer’s motor function. In this study, by reducing the 3D pattern to the 80% ratio, we developed a bra top that has a body shape correction effect as desired by new senior consumers, and a clothing pressure that does not impede physical activity or physiological function.

Finally, to verify the level of adequacy of this study’s developed bra top in reflecting new senior women’s requirements and suiting their body form, two market-available bra tops were selected and evaluated for comparison. In the appearance evaluation conducted by experts, the study-developed product was evaluated the most positively, followed by brands B and A; furthermore, in the evaluation conducted through virtual fitting technology, the developed product was evaluated the most positively, followed by brands A and B. The appearance evaluation conducted by experts determined what aspects could be judged with the eyes—that is, how well the bra top covered the surface of the body, how close it was to the body form, and whether there were wrinkles caused by tightening or pulling. On the other hand, the evaluation through the virtual fitting technology calculated the contact surface collision between the virtual body and the clothing and expressed how much the clothing was physically deformed in terms of color or numerical value. For this reason, it is suggested that a slight difference occurred between the results of the expert appearance evaluation and the virtual fitting. This study’s developed bra top received higher scores than the other brands both for the appearance evaluation and the virtual fitting evaluation. Sun et al. [40] revealed that the relative error between the results of the fitting test on real subjects and the results of the fitting test using virtual fitting simulation was within 10%, indicating that virtual fitting technology is suitable as a tool to evaluate bra’s fit. They measured breast deformation and pressure distribution by region, and as a result, the region under the shoulder strap received the highest pressure compared to other regions. Likewise, in the results of this study, it was found that the factor that most influenced the fit of the bra top was the clothing pressure that occurs at the shoulder. Kaixua et al. [44] measured clothing pressure using CLO 3D software to identify factors that affect the wearability of pants. They measured the stress value to calculate the tolerance range of clothing pressure for each part of the body. In this study, not only the stress value, but also the strain map and fit map of the garment were analyzed for the objective evaluation of the garment fit. Lee and Kim [68] analyzed research trends related to the fit of clothes and reported that previous studies mainly used methods for experts to evaluate the appearance of experimental clothing worn by subjects, or for subjects to evaluate the fit of their clothes. However, they claimed that such evaluations are difficult to verify objectivity because they rely on the subjective senses of evaluators and various attempts have been made to introduce new evaluation methods with objectivity in recent years. One of them is to evaluate the clothing fit using a virtual fitting system, which has the advantage of reducing time and cost because it does not go through the process of producing an actual garment, and above all, obtaining objective data through a scientific approach [69,70]. In the current work, in line with the trend of these studies, we evaluated the clothing fit using virtual fitting techniques in addition to an expert’s appearance evaluation.

This study set the optimal pressing rate at 80%, and the product was developed by applying even pressure to all the pattern pieces. However, this study has limitations in that it was not possible to set the optimum pressing ratio for each piece of the pattern and that various clothing materials were not used when manufacturing a bra top. Any follow-up study should find an appropriate clothing pressure for each body part by applying different reduction rates for each piece of the bra pattern; furthermore, follow-up studies should establish the appropriate pressurization value according to the clothing material.

In this study, a bra top was developed based on a virtual human body corresponding to the medium size of a new senior woman. However, to improve the fit of new senior consumers, it is necessary to create a virtual human body that quickly and accurately reflects not only the medium size but also the body types of various consumers. In addition, since this study was conducted on elderly Korean women in their 50s, attention should be paid to interpreting the results. In the future, if follow-up studies are conducted on a larger number of samples and variously classified groups, it is expected to contribute to improving the fit of bra top products.

Moreover, this study allowed for the bra top fit to be evaluated in two ways: measured through expert appearance evaluation and virtual fitting technology. However, this study has a limitation in that the fitting test was not performed on real humans. By performing a fitting test on an actual human body, a follow-up study should develop a method that comprehensively evaluates the clothing fit by revealing the clothing pressure value extracted from the clothing pressure meter, the clothing pressure that the subject feels, and the relationship between the expert appearance evaluation and the virtual fitting results. Regarding any follow-up studies related to virtual fitting, it is necessary to prepare certain criteria for evaluating the fit according to the range of the pattern deformation rate, and further, to develop a scale that integrates various parameters that represent the virtual fitting result into one scale. The development of such technology is expected to improve consumer satisfaction by helping consumers choose clothes in an online personalized market in the future.

## 5. Conclusions

In the past, the bras worn by women every day focused on protecting the body or correcting breast shape; however, the focus of bra design has now been shifting toward actively managing health. One of the most important variables related to health in clothing is clothing pressure. In particular, how to distribute the clothing pressure generated from the shoulders when designing bra products is a very important issue for researchers to address. In this study, when designing a bra, it was found that pressure distribution can be improved by reflecting the structural features of the garment, such as the position of the shoulder strap, the width of the shoulder strap, and the design line in the design. Successful aging is directly related to mental and physical health; in addition, reducing unhealthy clothing pressure helps maintain health, which improves the quality of life of new senior women.

In the field of apparel science research, when evaluating the clothing fit, most studies have relied on visual and qualitative evaluations. However, with the recent development of virtual fitting technology to represent clothing appearance through a three-dimensional simulation, it has become possible to evaluate the fit of clothing based on objective data. The apparel industry is beginning to develop a deep interest in 3D clothing fit because it could facilitate more efficient and effective decision-making in product development and quality control. This study used virtual fitting technology as a tool to measure clothing pressure and verified that 3D virtual fitting technology can be an adequate mechanism for replacing existing evaluation methods that are based on emotional judgment.

Recently, clothing manufacturers have shifted from mass production to mass customization systems to actively and quickly respond to individual customers’ body shape, body size, design preference, and so on, as consumers demand to express their individuality. In the future, the clothing design is expected to become more personalized, with customers directly participating in the design process. Virtual human body models and digital clothes based on wireframes are expected to help consumers secure the stability of their choice of clothing products in virtual reality and respond to various consumer needs. Based on ICT technology, allowing a human to try on a product in a virtual space and purchase that product will further accelerate the customized and personalized production service. Meanwhile, thanks to the development of high-tech technologies and increased interest in health, research to develop a healthcare apparel product that combines information technology with apparel products is being actively conducted. Healthcare clothing is clothing with cleanliness, comfort, and protection functions to maintain, enhance, or restore the health of the human body and to add comfort to daily life. To develop such healthcare clothing, it is necessary to analyze an individual’s body shape and develop appropriate clothing. In this study, representative models and digital patterns of new senior women were produced through 3D modeling and flattening techniques. The findings could provide basic data to design healthcare clothing products.

## Figures and Tables

**Table 1:** Human body dimensions of the target group (size 80A) (Unit: mm, kg, °).

Category		New Senior Woman (50–59 Year) *n* = 42			
		M	SD	Min	Max
height	stature	1563	55.8	1483	1635
	axilla height	1159	48.4	1044	1252
	bust height	1111	45.7	1011	1214
	under bust height	1057	46.7	966	1160
circumference	chest circumference	895	21.3	856	949
	bust circumference	898	17.6	872	929
	under bust circumference	798	13.9	785	821
length	posterior shoulder length	390	18.8	365	410
	interscye fold, back	363	17.0	327	401
	anterior–posterior axillary fold length	167	8.8	140	196
	interscye fold, front	331	14.5	287	357
	bust point breadth	176	8.9	151	200
	neck shoulder point to breast point	260	14.6	247	330
etc.	body weight (kg)	56.0	4.1	48.7	67.7
	body mass index (BMI)	23.0	1.7	20.5	25.3
	shoulder slope (R) (°)	23.0	3.2	21.0	26.0
	shoulder slope (L) (°)	20.4	2.4	18.0	25.0
body axis angle	neck angle (°)	85.2	4.3	77.7	92.7
	upper body axis angle (°)	96.5	4.1	89.1	102.4
	lower body axis angle (°)	94.7	0.8	93.1	96.2

M = Mean; SD = Standard deviation..

**Table 2:** Results of error verification of the 3D molded bra cup (Unit: mm, %).

	2D Flat Pattern	Molded Bra Cup	Relative Error (%)
A (upper side)	141.30	152.00	7.57
B (lower side)	231.20	236.00	2.08
C (cup center width)	173.10	170.00	−1.79

**Table 3:** Two bra tops and developed product selected for comparison.

	Brand A	Brand B	Developed Product
Product picture	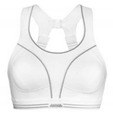	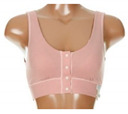	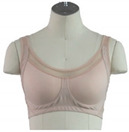
technical drawing	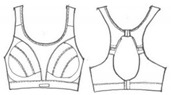	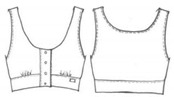	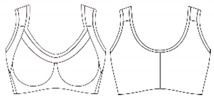

**Table 4:** Results of the appearance evaluation by experts.

Evaluation Questions		Brand A		Brand B		Developed Product	
		M	SD	M	SD	M	SD
breast area	1. Does the upper side cup cover the breast?	3.57	0.21	3.30	0.76	4.60	0.46
	2. Does the outside of the cup follow the breast?	2.73	0.25	2.97	0.39	4.73	0.22
	3. Does it cover the chest as a whole?	2.77	0.31	2.97	0.52	4.67	0.30
shoulder area	4. Is the strap’s press on shoulder area appropriate?	2.93	0.09	4.07	0.19	4.03	0.58
	5. Is the strap placed properly?	1.93	0.34	4.27	0.25	4.40	0.40
back area	6. Is the underarm area on the back covered?	1.43	0.39	4.03	0.42	4.07	0.41
	7. Is the pressing degree of the lower side of the side wing appropriate?	1.50	0.38	4.33	0.41	4.27	0.27
	8. Is the back side comfortable overall?	1.50	0.22	4.40	0.49	4.37	0.31
M		2.30	0.27	3.79	0.43	4.39	0.37

5 Point Likert Scale: 1 Strongly disagree, 2 Somewhat disagree, 3 Neither disagree nor agree, 4 Somewhat agree, 5 Strongly agree, M = Mean; SD = Standard deviation.

**Table 5:** Results of virtual fitting by Strain Map.

Category	Strain Map
brand A	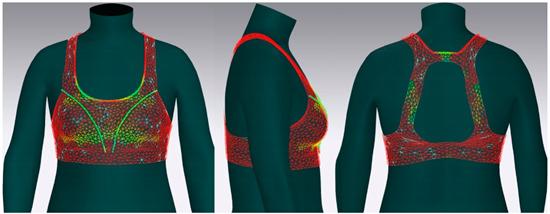
	Average pressure in the shoulder area: 21.28 kPa Average strain in the shoulder area: 151.89%
brand B	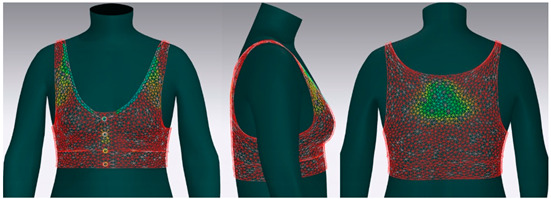
	Average pressure in the shoulder area: 71.91 kPa Average strain in the shoulder area: 176.39%
developed product	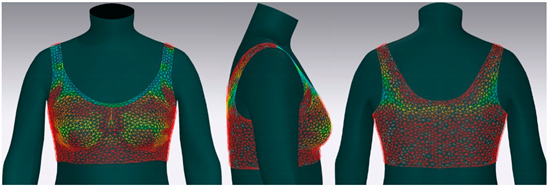
	Average pressure in the shoulder area: 11.94 kPa Average strain in the shoulder area: 104.10%

Strain Map appears in eight colors: A case in which there is significant strain is expressed in red, and a case in which there is no strain is expressed in blue.

**Table 6:** Results of virtual fitting by Fit Map.

Category	Fit Map
brand A	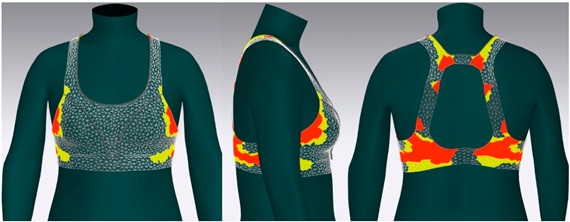
	Cannot wear: 17.0% Tight: 30.1%
brand B	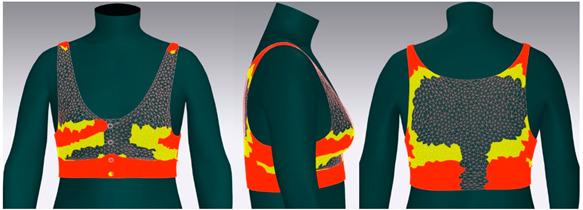
	Cannot wear: 39.7% Tight: 56.7%
developed product	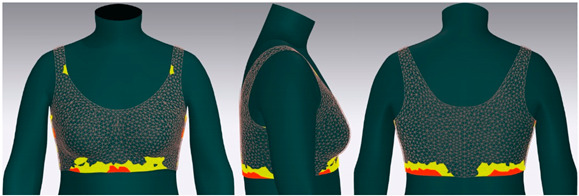
	Cannot wear: 2.9% Tight: 10.9%

Cannot Wear appears in red, which indicates a not wearable area, Tight appears in yellow, which indicates a slightly tight area.
